# Preprocessing by a Bayesian Single-Trial Event-Related Potential Estimation Technique Allows Feasibility of an Assistive Single-Channel P300-Based Brain-Computer Interface

**DOI:** 10.1155/2014/731046

**Published:** 2014-07-07

**Authors:** Anahita Goljahani, Costanza D'Avanzo, Stefano Silvoni, Paolo Tonin, Francesco Piccione, Giovanni Sparacino

**Affiliations:** ^1^Department of Information Engineering, University of Padova, Via Gradenigo 6/B, 35131 Padova, Italy; ^2^I.R.C.C.S. San Camillo Hospital Foundation, Via Alberoni 70, 30126 Venice, Italy

## Abstract

A major clinical goal of brain-computer interfaces (BCIs) is to allow severely paralyzed patients to communicate their needs and thoughts during their everyday lives. Among others, P300-based BCIs, which resort to EEG measurements, have been successfully operated by people with severe neuromuscular disabilities. Besides reducing the number of stimuli repetitions needed to detect the P300, a current challenge in P300-based BCI research is the simplification of system's setup and maintenance by lowering the number *N* of recording channels. By using offline data collected in 30 subjects (21 amyotrophic lateral sclerosis patients and 9 controls) through a clinical BCI with *N* = 5 channels, in the present paper we show that a preprocessing approach based on a Bayesian single-trial ERP estimation technique allows reducing *N* to 1 without affecting the system's accuracy. The potentially great benefit for the practical usability of BCI devices (including patient acceptance) that would be given by the reduction of the number *N* of channels encourages further development of the present study, for example, in an online setting.

## 1. Introduction

Brain-computer interfaces (BCIs) are cutting-edge systems aimed at identifying subjects' intention from measurements of brain activity [1–3]. A major clinical challenge in BCI research has been to develop systems capable of restoring communication in those people who, because of brainstem strokes, cerebral palsies, brain/spinal cord injuries, or progressive neurodegenerative diseases (such as amyotrophic lateral sclerosis, ALS), have lost the control of nearly all voluntary muscles but still retain cognition and sensation [4, 5].

Noninvasiveness, high temporal resolution, and low encumbrance are among the reasons why EEG-based BCIs are considered particularly appealing for the development of systems intended to be finally used at patients' home [3, 6]. The literature is huge and here is sufficient to mention that in severely paralyzed or disabled patients EEG-based BCIs have been successfully operated by exploiting slow cortical potentials [7, 8], sensorimotor *μ* and *β* rhythms [9–12], the P300 component of event-related potentials (ERPs) [13–20], and the electrical activity associated with semantically conditioned mental responses [21].

While some of the above-cited control signals may require long trainings for being reliably produced accordingly with users' intention, for example, several weeks in [7, 10], the P300 requires only focused attention and a suitable stimulation scheme for being generated, approximately 300 ms after a rare or task-relevant (target) stimulus [22]. Since the ERP is embedded in background EEG activity, the target stimulus has to be repeated several times in order to detect the P300, often from the average of *M* EEG epochs aligned with the stimulus onset [16, 19]. However, reliable generation and detection of the P300 with as few as possible stimulus repetitions (trials) is needed for obvious practical reasons. To this aim, BCI investigators have studied ad hoc stimulation schemes [20, 23–25], optimal channel sets [14, 26], channel selection methods [27, 28], classification algorithms [29–32], and spatial filtering approaches [18, 33–35]. These efforts led to the development of P300-based BCIs reliably operable by a very small number of repetitions (~4 in [20] with *N* = 8 channels), and, in few cases, even to single-trial systems, obtained, for example, by spatially filtering data from *N* = 5 recording channels [13, 17].

Yet, speed and accuracy are not the only concerns in BCI research. Particularly in clinical applications, user's physical condition may render simplicity of use and setup even more important than a theoretical 100% accuracy [36], as witnessed, for instance, by the fact that a patient involved in the study reported in [16] decided to withdraw from the investigation because attaching and maintaining too many electrodes was considered unacceptable. This is, indeed, a general concern shared by BCI users and their caregivers [37] and, although six to eight channels were believed to be optimal for classification accuracy in P300-based BCIs [26, 30], reduction of channels is advocated [16].

In this paper, we consider the challenge of reducing to one the number *N* of channels in a single-trial P300-based BCI. Specifically, as a preliminary proof of concept of the feasibility of an assistive single-channel (SC) BCI, we assess the hypothesis that the performance of the multichannel (MC) BCI system documented in [13], with *N* = 5, is preserved when only one channel is employed but a Bayesian ERP estimation technique [38, 39] is used to preprocess the signal. For such a scope, an offline comparison is made by using the data collected in 21 ALS patients and 9 healthy controls. Results will show that, in terms of classification accuracy, the performance of the SC BCI is not significantly different from that of the MC BCI, published in [13].

The paper is organized as follows. In Section 2, after a brief review of experimental protocol and reference MC BCI used in [13], the algorithms of the SC BCI prototype are described. Accuracy of the SC and MC systems is assessed and compared in Section 3. Comments on results, practical challenges, limits of the study, and margins for further investigations are reported in Section 4. Some conclusions end the paper in Section 5.

## 2. Materials and Methods

### 2.1. Experimental Protocol and Database

**Figure 1:**
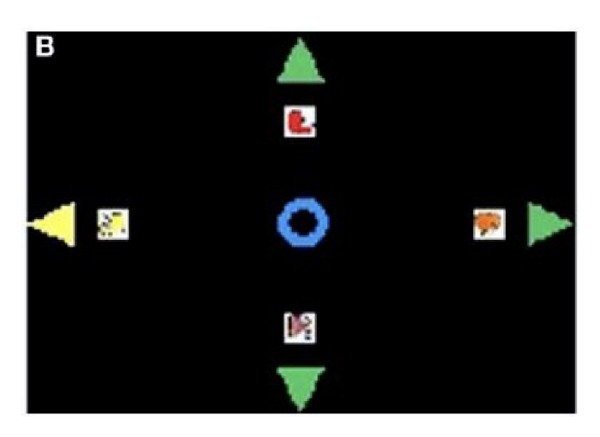
Graphical interface of the reference P300-based BCI [13]. The four icons represent four different basic needs. The flashing arrow on the left side is an example of stimulation.

Data utilized in the present study for offline analyses are those recorded during the online BCI sessions described in [13] from 21 ALS patients (aged 55.6 ± 14.3 years), in early and middle stages of the disease (32.2 ± 6.7 ALSFRS-R score [40], 47 ± 32 months from the disease onset), and 9 healthy controls (aged 54 ± 18.8 years). We refer the reader to [13] for details. Briefly, users were faced with a monitor with four images at its borders, representing four basic needs, for example, being hungry, and a circle in the center, as shown in Figure 1. The stimulation consisted of consecutive blocks of randomized flashings of four arrows (upward, rightward, downward, and leftward), each pointing to the direction of one image. By focusing on the flashing of the arrow pointing to the desired image (target stimulus) and ignoring the others (nontarget stimuli), a P300 component was elicited. EEG was recorded from four channels, that is, Fz, Cz, Pz, and Oz, and the electrooculogram (EOG) was recorded from two electrodes placed laterally and below the left eye. All electrodes were referenced to the left earlobe. Signals were amplified by a SynAmps (NeuroSoft, Inc.) amplifier, band-pass filtered between 0.15 and 30 Hz, digitized with a 16-bit resolution, and sampled at 200 Hz. After each flashing, a detection of a P300 activity from the measured EEG determined the movement of the circle by one step towards the direction of the flashed arrow and four consecutive steps in the desired direction were needed to reach the image. The time interval between two consecutive flashings, that is, the inter stimulus interval (ISI), was 2.5 s. Each BCI session started with the circle at the center of the screen and ended when the user reached the desired image or a time-out occurred (defined below for testing sessions only).

Sessions were distributed over five days. In the first day, eight sessions were carried out to collect data for initial calibration tasks. In particular, target and nontarget EEG data were recorded with an automatic feedback; that is, after each target flashing, the system automatically moved the circle towards the target image and, after each nontarget flashing, the system kept the circle still. In each automatic-feedback session, the number of flashings varied from a minimum of 13 to a maximum of 16, depending on when the target arrow was flashed in the fourth randomized block. In each of the subsequent testing days, denoted as T1, T2, T3, and T4, four sessions with feedback based on the identification of the P300 by means of the classification algorithm were carried out. Each testing session ended when the user reached the desired image or after a maximum of 92 flashings, corresponding to a time-out of 3 minutes and 50 seconds.

A classification error in correspondence with a nontarget stimulus determined the movement of the circle towards the wrong image, whereas a classification error in correspondence with a target stimulus determined the lack of a movement towards the desired image.

### 2.2. The Reference Multichannel (MC) BCI

**Figure 2:**
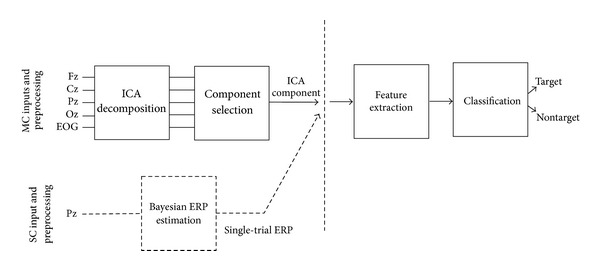
Functional blocks of the reference MC BCI and of the simulated SC prototype. MC (solid lines) and SC (dashed lines) preprocessing blocks are highlighted on the left side.

EEG raw epochs, starting 500 ms before and ending 1000 ms after each flashing, were extracted and baseline was corrected to the mean of prestimuli data, resulting in *n*_pre_ = 100 prestimulus and *n*_post_ = 200 poststimulus samples. Functional blocks of the reference multichannel (MC) BCI [13] are graphically illustrated in Figure 2. Briefly, in correspondence with each flashing, raw epochs from *N* = 5 channels, that is, Fz, Cz, Pz, Oz, and EOG, fed a single-trial ICA decomposition block that produced five independent components. One of the components was, then, selected and used to extract the information (features) supplied to the classifier to take the decision about whether the stimulus that produced the signal was target or nontarget. Seventy-eight features were computed for each epoch and comprised, for example, latencies and values of minimum and maximum peaks, power of the signal in 200 ms windows, and wavelet coefficients [13, 17]. Feature vectors were classified by means of a support vector machine (SVM) classifier with a radial basis function kernel [41].

As explained in more detail in [13], calibration tasks were performed at the beginning of each testing day using data collected during all preceding BCI sessions. Specifically, based on calibration data, for each subject, the ICA demixing matrix and the index of the component to be selected were determined offline by means of the algorithms described in [42, 43], respectively. Moreover, parameters needed for running the SVM classifier were derived by a cross-validation procedure on sets obtained by repeatedly splitting calibration data in 80%/20% fractions.

### 2.3. The Single-Channel (SC) BCI Prototype

The single-channel (SC) BCI prototype that we simulate and assess offline in the present paper is obtained by replacing the preprocessing steps of the MC BCI (blocks with solid lines in the left portion of Figure 2), which exploits 5 inputs, with a single-trial ERP estimation algorithm operating only on the Pz channel (block with dashed lines in Figure 2). Single-trial ERPs are estimated through the Bayesian approach extensively described in [38], which also admits further sophistications, recently documented in [39, 44], though not usable in real time and thus unsuited to the BCI setting.

Briefly, the method performs an ad hoc smoothing of each EEG raw epoch by exploiting, in a Bayesian setting, prior knowledge on the smoothness of the unknown ERP, described as *m* discrete integrations of a white noise with variance *λ* ^2^, and on the autocorrelation of the background EEG (noise), obtained from an AR model of order *p* with coefficients and variance parameter *σ* ^2^ identified from the prestimulus data of each epoch. In our application, *m* = 2, while *p* is determined, according to the final predictor error (FPE) criterion, epoch by epoch. *λ* ^2^ being unknown, the ratio *γ* = *σ* ^2^/*λ* ^2^, which determines the single-trial smoother, is determined, epoch by epoch, by the popular discrepancy regularization criterion [45]. An example of application of the single-trial ERP estimation to our BCI data is described in Section 3.1.

After Pz data preprocessing, the same features defined and employed in the reference MC BCI are extracted (from the estimated single-trial ERPs) and, finally, classified by an SVM classifier with the same type of kernel. In order to allow a fair comparison between SC and MC, available datasets were processed offline by the SC BCI in the same way as in the online MC BCI. Specifically, for each subject, before processing single-trial data from each testing day, calibration tasks, for example, classifier training, were performed based on Pz datasets from all preceding sessions. This was done to obtain, for each subject and testing day, accuracy comparable to that obtained by the MC BCI and assess the role of the proposed preprocessing approach on the system's performance.

*Remark*. The determination, epoch by epoch, of *γ* can be computationally expensive (see [46]). While this is not an issue for offline calibration tasks, it can affect online operability. For this reason, in the present paper, for each testing session, instead of optimizing *γ* for each epoch, we utilize a fixed γ* predetermined from calibration data as the median of the set of *γ* values “optimally” tuned, for all target and nontarget calibration epochs, by the discrepancy criterion.

## 3. Results

### 3.1. Output of the New Pre-Processing Step

**Figure 3:**
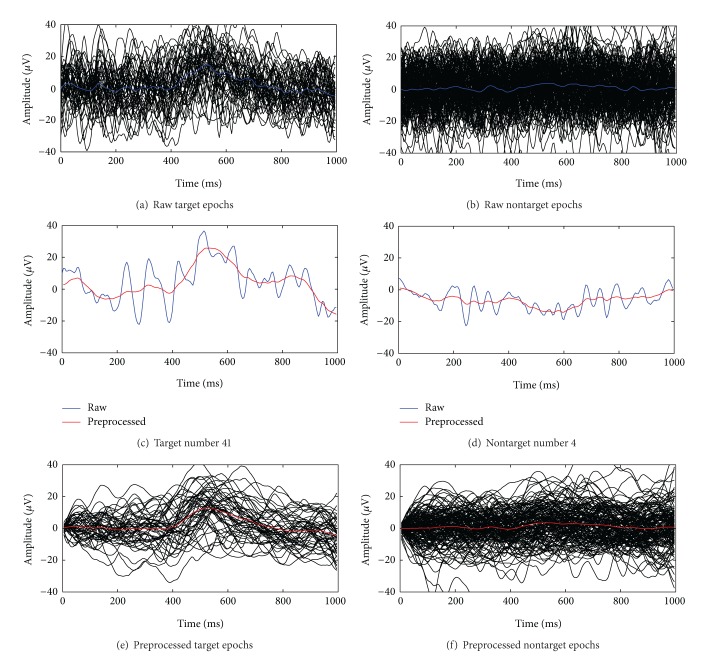
Preprocessing results for a representative ALS patient. Raw target and nontarget epochs collected from P8 in T4 are shown in (a) and (b), respectively, together with their average, shown as solid blue lines. In (c) and (d) two representative raw target and nontarget epochs (blue curves) are superimposed to their denoised versions obtained by the Bayesian preprocessing (red curves). In (e) and (f) signals obtained by preprocessing target and nontarget epochs in (a) and (b), respectively, together with their averages (red curves), are shown.

Before investigating the performance of the BCI system, it is useful to show an example of application of the single-trial estimation technique to our data.  Figure 3 reports, in panels (a) and (b), respectively, single-trial target and nontarget raw epochs recorded from Pz in patient P8 during the testing day T4. The blue curves drawn in the same panels are the averages of target and nontarget raw epochs, respectively. Panels (c) and (d) display results of the new preprocessing step (red curves) for one representative target and one representative nontarget raw epoch (blue curves). Finally, the black curves in panels (e) and (f) are the preprocessed versions of all curves in panels (a) and (b), respectively, and the red curves are their averages. As visible from the blue curves in panels (a) and (b), target raw epochs are characterized by an average positive deflection that is not present in nontarget epochs. The deflection, which takes place at around 500 ms, is the P300 component of the ERP. The red curve in panel (c) shows that, at the single-trial level, the considered preprocessing smoothes away spurious oscillations and produces a signal in which the P300-related activity is more evident. As far as nontarget epochs are concerned, panel (d) confirms that, as expected, the proposed preprocessing yields a signal that is quite flat, reflecting the absence of a P300-related activity. Finally, panels (e) and (f) show how the extracted activity varies from epoch to epoch, with average activities (red curves) similar to the ones of raw epochs.

**Figure 4:**
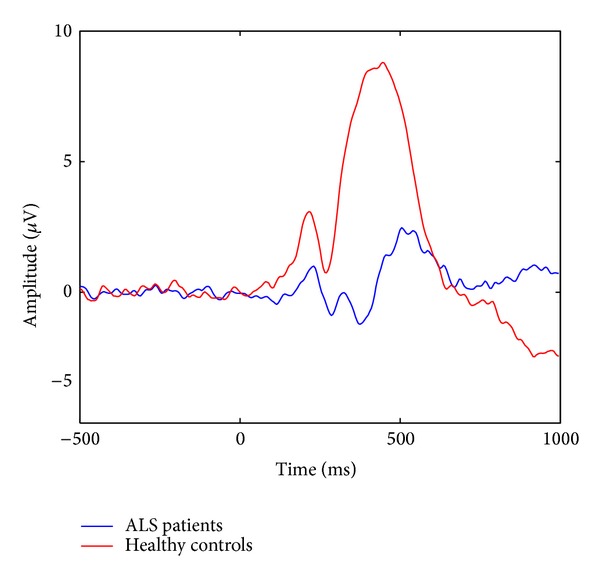
Average of target epochs collected from Pz during all testing days for ALS patients (red curve) and healthy controls (blue curve).

Similar comments could be drawn from the results obtained in the other patients and in healthy subjects, irrespectively of ERP interepoch variations and general group differences, visible, for example, from the grand averages depicted in Figure 4, which show a lower and slightly delayed P300 for patients with respect to controls. Indeed, according to the comprehensive simulation studies reported in [38, 39], the accuracy of ERP estimates depends on the signal-to-noise ratio (SNR) and not on the specific shape of the ERP.

### 3.2. Assessment of Classification Accuracy: SC versus MC

Classification accuracy obtained by feeding the SC prototype with the input data described in Section 2.1, together with the reference accuracy published in [13], is reported in Tables 1 and 2 for ALS patients and controls, respectively. Results are grouped by testing day and, for brevity, accuracy by the MC BCI of [13] and by the SC BCI prototype is denoted as “MC accuracy” and “SC accuracy,” whereas labels P1,…, P21 and H1,…, H9 are used for patients and controls, respectively.

**Figure 5:**
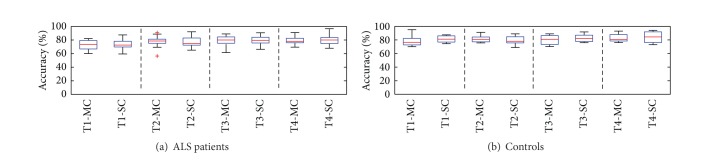
Distributions of accuracy achieved by MC and SC systems in T1,…, T4 for ALS patients (a) and controls (b). Labels T*j*-MC and T*j*-SC for, *j* = 1,…, 4, denote accuracy achieved in testing day T*j* by MC and SC systems, respectively.

Numerical results in Tables 1 and 2 are graphically represented in Figure 5, where a series of box-plots are reported in two panels related to ALS patients (panel (a)) and healthy controls (panel (b)), respectively. Each box-plot is based on values from one column of the tables, that is, on the accuracy achieved in a specific testing day, T_*i*_, *i* = 1,…, 4, and by a specific system, MC or SC. The lower and upper edges of the rectangles are drawn in correspondence with the 25th and 75th percentile of accuracy, respectively, and the red lines represent the median accuracy.

As shown by the plots, in all testing days the median accuracy yielded by the two systems is very close both for patients and for controls. Actually, based on values in Tables 1 and 2, it can be assessed that differences between medians are below 5% and, whereas the average performance in T1 and T2 is slightly higher when the MC BCI is employed, the inverse happens in T3 and T4, where the SC prototype yields slightly higher medians for both groups, for example, 81% versus 78% and 84,5% versus 80,7% for patients and controls, respectively, in T4. The plots in Figure 5 also show that for all testing days the distributions of accuracy around the medians mainly overlap for the two systems, without a clear predominance of one system over the other. For instance, in T4 the accuracy achieved by ALS patients is concentrated between 76,1% and 82,7% for the MC BCI and between 74,9% and 83,7% for the SC prototype. Similarly, for healthy controls, the ranges are 78,5–87,9% and 76,3–91,9%, respectively. In order to validate these graphical evidences, MC accuracy and SC accuracy were compared by means of a statistical test. Specifically, both for patients and for controls, a Wilcoxon paired test was performed on accuracy from each testing day. None of the tests revealed statistically significant differences, confirming that, thanks to the proposed preprocessing, users' intention can potentially be decodable from only one channel with the same accuracy achieved with the reference *N* = 5 channels BCI. As a final remark, in terms of best cases, it is interesting to note that maximum accuracy achieved by ALS patients in each testing day by means of the SC prototype, that is, 87.5%, 92.2%, 90.7%, and 96.7%, in T1, T2, T3, and T4, respectively, besides being impressively high from the first day, slightly outperforms that achieved by the MC BCI, that is, 82.2%, 90.9%, 89%, and 91%, with the best improvement of 5.7% obtained in T4. The same happens for healthy controls in T3 and T4, where the SC prototype yields maximum accuracy of 91,8% and 94,1%, respectively, versus the 89% and 93,1% of the MC BCI. It is worthy to note that all users only participated in the five-day experimental protocol described in Section 2.1.

## 4. Discussion

Offline analysis on data collected in 21 ALS patients and 9 controls indicates that the proposed Bayesian preprocessing technique yields signals with enhanced SNR well evidencing the presence or absence of P300-related activity. Accuracy of the SC prototype was found to be not statistically different from that of the MC BCI, confirming that no performance degradation was caused by the reduction of channels from *N* = 5 to *N* = 1.

Remarkably, results about SC accuracy, reported in Section 3.2, were obtained without any strategy to individually select the single channel. Interesting insights in this regard may be obtained from an accurate inspection of the outcomes in some specific cases (details not reported for the sake of brevity of the paper). For instance, in those cases in which SC performed worst, we observed that Pz did not show an evident P300 activity and that an improvement in accuracy could be achieved by using data from Cz, for example, from 67.9% to 80.7% for patient P11 in T4. Similar examples were found that highlighted the benefit of an optimized electrode positioning in SC systems.

As far as communication speed is concerned, the 2.5 s ISI of the considered four choices paradigm yields an upper bound of 12 bit/min for the information transfer bit rate (TBR), computed as in [17]. Interestingly, this value is almost achieved by P15 in T2, where the SC prototype allows a communication of 11.2 bit/min.

Limits of the present study include the fact that results were obtained in an offline setting, where adaptation mechanisms that take place when the user receives the feedback to his/her mental activity cannot be reproduced. Therefore, an online implementation of the SC prototype will be needed to validate and confirm the results obtained in the simulated environment of this paper. Another potentially critical point concerns the ISI, which should be reduced in order to increase communication speed. Obviously, short ISIs, for example, the 400 ms proposed in [14] for a six-icon paradigm, determine a possible spread of the target P300 activity in epochs corresponding to nontarget stimuli. Proper investigations would be needed in this case to assess the capability of the classifier to predict users' intention from the activity estimated by the Bayesian technique. In any case, a short ISI would require improving numerical implementation of the Bayesian preprocessing algorithm. In particular, in place of the matrix-vector approach [38] used also in the present paper, methods implementing Wiener filtering by* Z*-transforms and spectral factorization techniques [47] should be chosen.

Finally, it is worth mentioning that the number of ALS datasets available for the present work, that is, 21, is quite high with respect to commonly published BCI investigations. In perspective, this adds to the potential clinical interest of the study, because, sometimes, results obtained in healthy subjects do not reflect those of patients [48].

## 5. Conclusions

The study presented in this paper shows that a preprocessing by a sophisticated single-trial ERP estimation technique can potentially allow driving a P300-based BCI by a single channel. This can be of high impact in BCI clinical applications, where simplicity of use and setup can be even more important than 100% of accuracy and reduction of the number *N* of channels has been solicited [16]. Moreover, a reduced number of channels would also have an impact on power consumption in wireless EEG caps.

As future developments of the present study, besides validating results in an online setting and with diverse stimulation schemes with variable ISIs, further research will be aimed at better understanding of to what extent single-trial ERP estimation techniques may effectively contribute to improve P300-based BCI systems, for example, by optimizing the position of the single channel. A further boost to the practical usability of P300-based BCIs may be given by the use of a subcutaneous (minimally invasive) electrode such as that proposed in [49] for hypoglycemia detection.

## Figures and Tables

**Table 1:** Percentage accuracy achieved by ALS patients in testing days T1, T2, T3, and T4 by means of the MC BCI (MC acc.) and the SC BCI prototype (SC acc.).

	T1		T2		T3		T4	
	MC acc. (%)	SC acc. (%)	MC acc. (%)	SC acc. (%)	MC acc. (%)	SC acc. (%)	MC acc. (%)	SC acc. (%)
P1	73,5	72,6	73	78,4	78,3	81,7	77,9	81
P2	60,3	71,3	81,3	76,1	74,2	71,4	70,8	75,2
P3	63,4	69,2	90,9	69	80,6	70,5	87,2	74,1
P4	65	74,9	69,5	73,4	61,6	74,9	75,3	73,5
P5	80,2	78,8	80,6	70,6	89	83,7	82,1	82
P6	80,9	78,6	81,3	86,2	86,4	89,3	88,7	85,2
P7	72,9	80,7	76,2	75,4	74,7	79,2	72,7	80,1
P8	76,6	86,1	78,6	83,7	80,6	76,7	76,3	83,7
P9	80,4	78,6	78,4	90,2	79,1	88,7	84,4	83,9
P10	82,2	76,2	77,9	79	80,1	85,6	79,4	87,5
P11	82	72,3	78,8	78,5	74,7	76,5	85,2	69,7
P12	79,1	76,5	85,7	75,5	70,7	81,5	79,3	77
P13	77,6	77,6	86,4	86,3	88,4	83,1	91	96,7
P14	75,9	72,9	79	74	85,4	83,2	77,2	81,5
P15	67	87,5	79	95,1	79,2	90,7	80,3	89,7
P16	61,5	63,9	56,4	74,8	79,4	78,4	76,8	83,7
P17	70,6	70,4	75,3	76,6	80	74,2	71,4	70,9
P18	66,4	70,3	74,6	74,8	80,9	79,5	78	80
P19	78,1	82,5	88,8	85,3	84,3	85,9	69,6	84,9
P20	72,9	72,4	77,5	70	86,4	76,8	78,9	74,1
P21	72,7	72,3	69,5	76,9	75,5	82,9	77,4	79,3

**Table 2:** Percentage accuracy achieved by healthy controls in testing days T1, T2, T3, and T4 by means of the MC BCI (MC acc.) and the SC BCI prototype (SC acc.).

	T1		T2		T3		T4	
	MC acc. (%)	SC acc. (%)	MC acc. (%)	SC acc. (%)	MC acc. (%)	SC acc. (%)	MC acc. (%)	SC acc. (%)
H1	73,6	81,9	77,3	84,1	73,9	84,5	79,6	84,5
H2	73,3	81,2	77,5	86,5	86,6	91,8	78,6	91,9
H3	71,2	74,6	76,9	76,8	71,8	76	76,1	75
H4	85,9	87	80,8	89	86,3	86,7	89,4	94,1
H5	81,2	77,6	91,3	79,12	81	77,6	87,4	84,6
H6	76,4	79,6	83,1	80,6	81,9	79,8	78,2	73,1
H7	95,1	87,6	86,9	81,7	89	88,1	93,1	83,5
H8	70,1	75,8	83,6	68,9	76,1	77,9	83,5	76,7
H9	77,3	86,1	75,7	72,4	70,3	82	80,7	92
